# Clinical Significance of Conjunctival Microvascular Density in Diabetic Retinopathy: A Multimodal Correlation Study Based on Swept‐Source Optical Coherence Tomography Angiography

**DOI:** 10.1155/jdr/9076881

**Published:** 2026-01-24

**Authors:** Xiaoli Huang, Jiajia Yu, Wenjun Zou, Xiaoli Xiang, Hu Liu

**Affiliations:** ^1^ Department of Ophthalmology, The First Affiliated Hospital of Nanjing Medical University, Nanjing City, Jiangsu Province, China, njmu.edu.cn; ^2^ Department of Ophthalmology, Jiangnan University Medical Center, Wuxi No.2 People′s Hospital, Wuxi City, Jiangsu Province, China; ^3^ Department of Ophthalmology, Affiliated Changshu Hospital of Nantong University, Changshu City, Jiangsu Province, China

## Abstract

Diabetic retinopathy (DR) with media opacity presents a diagnostic challenge for retinal evaluation. This study investigated whether conjunctival microvascular assessment using swept‐source optical coherence tomography angiography (SS‐OCTA) can serve as a potential indicator of retinal pathology. We conducted a comparative study of 163 patients with diabetes (110 with DR, subdivided into 55 nonproliferative and 55 proliferative cases) and 49 age‐matched healthy controls. All participants underwent SS‐OCTA for conjunctival vessel density (VD) measurement and standard retinal OCTA for retinal VD and ganglion cell complex (GCC) analysis. Statistical correlations were performed to evaluate the relationship between the conjunctival and retinal parameters. Conjunctival VD showed a progressive reduction in DR severity, most prominently in the temporal region (70.7% in controls vs. 55.6% in patients with proliferative diabetic retinopathy [PDR]). Temporal conjunctival VD correlated well with retinal damage, and lower conjunctival density was linked to reduced retinal blood flow (*r* = 0.21–0.26) and thinner nerve layers (*r* = 0.22). No significant differences in VD were found between controls and patients with diabetes without DR, suggesting a specificity for retinopathic changes. SS‐OCTA assessment of conjunctival VD may provide clinically useful information regarding the retinal status in patients with DR with compromised fundus visualization. This approach is a practical alternative when traditional retinal imaging is obstructed by media opacities.

## 1. Introduction

Diabetic retinopathy (DR) is a leading cause of visual impairment worldwide, and its progression is characterized by retinal microvascular damage and neuroretinal degeneration [1]. Early detection and monitoring are critical for preventing vision loss; however, clinical assessment is often hindered in patients with media opacities such as cataracts or vitreous hemorrhage, which obscure fundus visualization [2]. While fluorescein angiography (FA) and optical coherence tomography angiography (OCTA) are the gold standards for evaluating retinal vasculature, their utility is limited in cases where optical media clarity is compromised [3].

The conjunctiva, a readily accessible vascular bed, shares embryological and physiological similarities with the retinal vessels, making it a plausible surrogate for studying microangiopathy in DR. The association between conjunctival and retinal circulation is not hypothetical; existing literature has confirmed their pathological and functional homology across multiple dimensions.

Anatomically, the systems originate from the embryonic ocular vascular network. The conjunctival arterial supply shares a homologous blood flow regulation pathway with the retinal vascular network. Both conjunctival vessels and retinal vessels belong to the terminal portion of the microcirculation [4]. The conjunctival arterial supply primarily originates from the anterior ciliary arteries and branches of the ophthalmic artery, with the accompanying veins forming an extensive vascular network that is particularly vulnerable to both systemic disorders and ocular pathologies. The scleral vasculature, located beneath the conjunctiva, receives a dual blood supply: a deep vascular plexus derived from the anterior ciliary arteries and a superficial vascular network, with drainage occurring through the intrascleral venous plexus and superficial venous system into the anterior ciliary veins. Although superficial conjunctival and episcleral vessels can be directly visualized using conventional imaging modalities such as FA and slit‐lamp biomicroscopy, noninvasive quantitative assessment of these vasculatures at predetermined depths remains technically challenging. This limitation is particularly pronounced in scleral imaging because of the inherent opacity of the tissue, which typically obscures intrascleral vascular structures. OCTA may offer a novel solution to these long‐standing visualization barriers by enabling depth‐resolved quantitative vascular analysis [5].

In systemic diseases such as diabetes and hypertension, the widening of conjunctival microvascular diameter, reduction in vascular density, and progression of retinal lesions show a high degree of synchrony, suggesting the existence of a conjunctival–retinal microcirculation injury cascade [6–8]. Studies utilizing standard slit‐lamp photography with digital red‐free imaging have demonstrated an overall reduction in bulbar conjunctival vascular density among patients with diabetes, suggesting a potential correlation between decreased vascular area and DR severity [9]. Recent advances in swept‐source optical coherence tomography angiography (SS‐OCTA) have enabled the high‐resolution imaging of the conjunctival microvasculature, offering a potential alternative for assessing systemic and ocular microvascular health [10, 11]. Preliminary studies suggest that conjunctival vascular alterations may reflect systemic diabetic microvascular complications; however, their specific correlation with DR severity and retinal structural changes remains underexplored [12].

This study was aimed at characterizing the conjunctival microvascular density (vessel density [VD]) changes across DR severity stages using SS‐OCTA, evaluating the correlations between conjunctival VD and retinal microvascular and neuroretinal parameters, and assessing whether conjunctival VD could serve as a surrogate marker for retinal pathology in eyes with media opacities. By addressing these questions, we sought to provide a clinically feasible tool for DR monitoring in patients in whom traditional imaging modalities fail, ultimately bridging a critical gap in the management of advanced diabetic eye disease.

## 2. Materials and Methods

### 2.1. Ethics Statement

This study was approved by the Institutional Review Board of the Jiangnan University Medical Center, Wuxi No.2 People′s Hospital (Approval No. 2023‐Y‐198). The study was conducted in accordance with the Declaration of Helsinki, and informed consent was obtained from all participants.

### 2.2. Sample Size Estimation

A priori sample size calculation was performed using G∗Power 3.1.9.2 [13] for one‐way ANOVA comparing four groups (healthy controls, diabetes without DR, nonproliferative diabetic retinopathy [NPDR], and proliferative diabetic retinopathy [PDR]). With an effect size *f* of 0.3 (moderate effect based on preliminary data showing 15%–20% conjunctival VD differences between groups), *α* = 0.05, and power = 0.90, the analysis indicated a minimum requirement of 164 total subjects (41 per group). To address potential subject attrition and exclusion due to insufficient image quality, the sample size was increased by 15%, with a final target enrolment of approximately 47–48 subjects per group, providing > 90% power to detect significant intergroup differences in conjunctival microvascular parameters.

### 2.3. Study Design and Participants

This study enrolled 163 patients with diabetes who visited the Department of Ophthalmology at Jiangnan University Medical Center between February 2023 and August 2024, including 110 with DR. Based on fundus photography and FA findings, the participants were stratified into NPDR (*n* = 55 eyes) and PDR (*n* = 55 eyes) groups. In addition, 49 age‐ and sex‐matched healthy subjects were recruited as controls.

#### 2.3.1. Inclusion Criteria

1. Participants capable of cooperating with the required examinations;
2. All diabetic patients underwent both fundus fluorescein angiography (FFA) and OCTA within 1 week;
3. OCTA images meeting quality standards;
4. The eligible eye was selected as unilaterally qualified. For bilaterally qualified cases, the more severely affected eye (determined by FFA) was chosen, with the right eye being preferred for symmetrical cases.

#### 2.3.2. Exclusion Criteria

1. History of ocular surgery or trauma;
2. Other retinal pathologies, including but not limited to macular edema, uveitis, retinal vein occlusion, age‐related macular degeneration, glaucoma, or high myopia;
3. Systemic comorbidities: severe cardiac, pulmonary, hepatic, or renal dysfunction and hematologic disorders;
4. Scleral contact lens wearers;
5. Media opacity compromising image quality.

### 2.4. Retina OCTA Protocol

All patients were examined using a VG200D SS‐OCTA device (Microvision Technology Co. Ltd., Luoyang City, Henan Province, China). AngioRetina was selected as the scanning mode, and 3D‐PAR and DeepLayer artificial intelligence layering algorithms were applied to remove projection artifacts from the blood flow and B‐scan images. The scanning area covered a 9 × 9 mm^2^ area centered on the macula. The calculation boundary of the inner retinal vascular density was set from the inner limiting membrane to the inner nuclear layer/outer plexiform layer, including the superficial, middle, and deep capillary networks. The scan area was divided into four regions: (1) within 1 mm of the central concave area, (2) a circumferential region 1–3 mm from the center, (3) a circumferential region 3–6 mm from the center, and (4) a circumferential region 6–9 mm from the center.

Each region was further divided into four fan‐shaped regions: superior, inferior, nasal, and temporal, and the average VD was calculated.

### 2.5. Ganglion Cell Complex (GCC) Analysis

The GCC within the 6 × 6 mm^2^ macular center area was analyzed, and its mean and minimum thicknesses were measured. In addition, the GCC was divided into six sectors (superotemporal, superior, superonasal, inferonasal, inferior, and inferotemporal) for the regional thickness analysis.

### 2.6. Conjunctival Vascular Flow Analysis

For conjunctival vessel assessment, the most exposed nasal and temporal regions were scanned using the anterior segment (AS) angiography mode (Figure 1). A 6 × 6 mm^2^ scan area was used for both regions (scanning mode: AS angio 6 × 6 384 × 384*R* ^2^). The 3D‐PAR algorithm, combined with DeepLayer AI segmentation, was used to eliminate projection artifacts in both en face flow images and B‐scan slices (software release: 2.1.016). VD analysis was performed within a boundary extending from the superficial conjunctival layer to the anterior scleral stroma, and the mean VD was calculated.

Figure 1SS‐OCTA scanning interface for conjunctival vascular density measurement. (a) SS‐OCTA scan image of the conjunctival vascular. (b) Quantitative SS‐OCTA analysis of a 6 × 6 mm^2^ temporal bulbar conjunctival region in a study participant.

**Figure:**
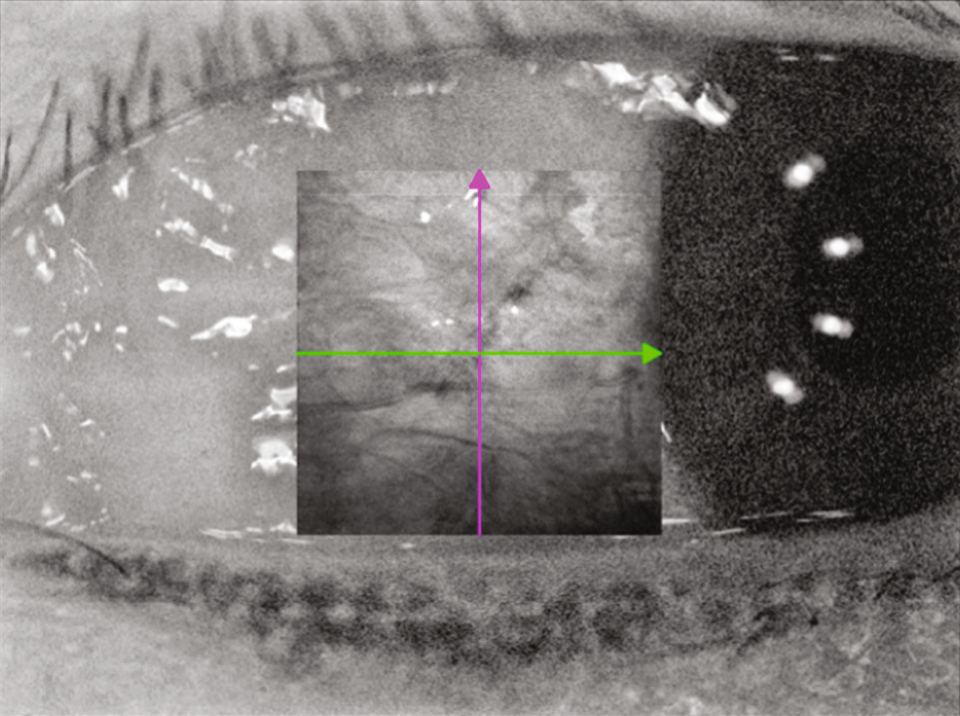
(a)

**Figure:**
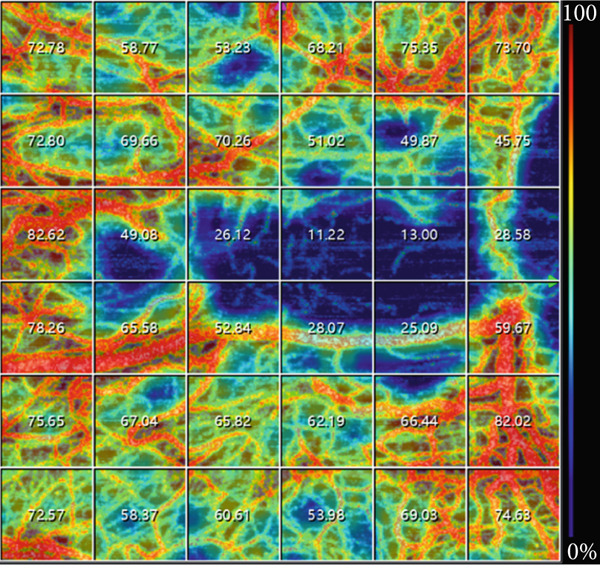
(b)

All OCTA scans were performed by the same trained technician and manually calibrated by two independent, experienced ophthalmologists to ensure measurement accuracy. Using this standardized approach, we comprehensively evaluated the vascular density and structural characteristics of the retina and conjunctiva.

### 2.7. Statistical Analysis

Statistical analyses were performed using SPSS software (Version 25.0; IBM Corp., Armonk, NY, United States). Heatmap visualization was performed using GraphPad Prism software (Version 9.0; GraphPad Software Inc., San Diego, CA, United States). Categorical variables are presented as frequencies and percentages (*n* [percentage]) and were analyzed using the chi‐square test. Continuous variables were tested for normality using the Shapiro–Wilk test. Normally or approximately normally distributed data were expressed as mean ± standard deviation and analyzed using an independent sample *t*‐test (two‐group comparisons) or one‐way ANOVA (multiple‐group comparisons), followed by Bonferroni post hoc tests for multiple comparisons. Non‐normally distributed data are reported as median (interquartile range) (M [P25, P75]) and compared using the Mann–Whitney *U* test (two groups) or Kruskal–Wallis test (multiple groups), with Bonferroni‐adjusted pairwise post hoc comparisons. Spearman′s correlation analysis was used to evaluate the relationships between the variables. Statistical significance was defined as a two‐tailed *p* value < 0.05.

## 3. Results

### 3.1. Baseline Characteristics

A total of 110 patients with DR were enrolled in this study (55 patients with NPDR). The demographic characteristics of the participants are shown in Table 1. The study revealed that there were no statistically significant differences in gender or age distribution among the diabetes mellitus (DM) group (without retinopathy), NPDR group, PDR group, and healthy control group. The duration of DM was not significantly different between the DM, NPDR, and PDR groups. However, a significant intergroup difference in best corrected visual acuity (BCVA) was identified among the four groups (*p* < 0.0001). Post hoc comparisons revealed no significant difference in visual acuity between the DM group and healthy controls (*p* = 0.670). The PDR group demonstrated the worst BCVA, with statistically significant differences observed among the DM, NPDR, and PDR groups (all *p* < 0.0001).

**Table 1:** Baseline demographic and clinical characteristics of the study population.

**Characteristic**	**Healthy controls (** **n** = 49**)**	**DM without DR (** **n** = 53**)**	**NPDR (** **n** = 55**)**	**PDR (** **n** = 55**)**	**Statistics**	**p** **value**
Sex (female/male)	25/24	22/31	26/29	26/29	*χ* ^2^ = 0.956	0.812
Age (years)	57.92 ± 10.68	61.96 ± 12.94	58.95 ± 11.42	58.35 ± 12.09	*F* = 1.253	0.292
Course of DM (years)	N/A	10.12 ± 7.14	12.05 ± 6.15	12.21 ± 6.32	*F* = 1.330	0.268
BCVA (LogMAR)	0.10 (0.10, 0.22)	0.22 (0.097, 0.30)	0.39 (0.28, 0.73)	1.00 (0.52, 1.09)	*H* = 103.194	< 0.0001

Abbreviations: BCVA, best corrected visual acuity; DM, diabetes mellitus; DR, diabetic retinopathy; NPDR, nonproliferative diabetic retinopathy; PDR, proliferative diabetic retinopathy.

### 3.2. Retinal Vascular Density of the Four Groups

Quantitative analysis revealed progressive retinal vascular attenuation with advancing DR severity of DR (Table 2). The 1–3 mm parafoveal region demonstrated the most pronounced changes. The nasal quadrant showed the greatest density reduction in PDR compared to controls, with significant pairwise differences across all disease stages. Notably, the temporal quadrant within 1–3 mm showed earlier involvement, with significant reductions already present in patients with NPDR compared to controls.

**Table 2:** Retinal vascular density parameters across study groups.

**Parameter**	**Healthy controls (** **n** = 49**)**	**DM without DR (** **n** = 53**)**	**NPDR (** **n** = 55**)**	**PDR (** **n** = 55**)**	**Statistics**	**p** **value**	**Post hoc comparisons**
Average within 1 mm of the central concave (%)	19.86 ± 8.31	15.69 ± 8.52	16.03 ± 10.53	13.92 ± 9.07	*F* = 3.514	0.016	c
Average 1–3 mm circumferential region (%)	62.73 ± 13.13	54.56 ± 15.07	51.75 ± 16.21	45.02 ± 17.12	*F* = 10.646	< 0.0001	b,c,e
1–3 mm circumferential region—Superior (%)	64.24 ± 15.69	59.69 ± 15.03	54.74 ± 18.57	47.12 ± 20.54	*F* = 8.041	< 0.0001	b,c,e
1–3 mm circumferential region—Temporal (%)	57.99 ± 14.22	51.38 ± 15.86	46.91 ± 18.46	43.26 ± 20.50	*F* = 6.321	< 0.0001	b,c
1–3 mm circumferential region—Inferior (%)	64.94 ± 15.15	54.23 ± 17.79	53.75 ± 17.95	47.67 ± 20.16	*F* = 7.735	< 0.0001	a,b,c
1–3 mm circumferential region—Nasal (%)	63.79 ± 14.21	52.98 ± 17.95	51.63 ± 17.83	42.07 ± 19.12	*F* = 12.398	< 0.0001	a,b,c,e,f
Average 3–6 mm circumferential region (%)	64.96 ± 8.88	58.70 ± 11.43	59.66 ± 10.68	57.52 ± 10.80	*F* = 4.705	0.003	a,c
3–6 mm circumferential region—Superior (%)	66.10 ± 11.04	60.50 ± 13.05	61.04 ± 12.51	59.44 ± 15.73	*F* = 2.442	0.066	N/A
3–6 mm circumferential region—Temporal (%)	55.93 ± 13.54	49.31 ± 14.80	49.84 ± 16.09	49.26 ± 17.44	*F* = 2.095	0.102	N/A
3–6 mm circumferential region—Inferior (%)	63.14 ± 11.60	57.86 ± 13.34	58.65 ± 13.47	56.21 ± 15.16	*F* = 2.320	0.077	N/A
3–6 mm circumferential region—Nasal (%)	74.65 ± 9.66	67.13 ± 13.98	69.09 ± 11.06	65.23 ± 13.49	*F* = 5.380	0.001	a,c
Average 6–9 mm circumferential region (%)	59.34 ± 6.66	55.25 ± 7.70	57.99 ± 8.27	57.17 ± 8.96	*F* = 2.032	0.111	N/A
6–9 mm circumferential region—Superior (%)	58.58 ± 11.05	54.63 ± 12.61	57.53 ± 9.87	56.33 ± 14.46	*F* = 0.867	0.459	N/A
6–9 mm circumferential region—Temporal (%)	39.59 ± 13.76	34.00 ± 13.99	39.84 ± 13.42	39.99 ± 14.75	*F* = 1.811	0.147	N/A
6–9 mm circumferential region—Inferior (%)	55.84 ± 10.72	53.22 ± 11.21	54.70 ± 11.70	54.89 ± 13.79	*F* = 0.360	0.782	N/A
6–9 mm circumferential region—Nasal (%)	83.29 ± 6.31	79.21 ± 9.45	80.13 ± 8.54	77.58 ± 9.24	*F* = 3.609	0.015	a

*Note:* Post hoc comparisons: a = healthy vs. DM, b = healthy vs. NPDR, c = healthy vs. PDR, d = DM vs. NPDR, e = DM vs. PDR, f = NPDR vs. PDR (all *p* < 0.05, Bonferroni′s correction).

Abbreviations: DM, diabetes mellitus; DR, diabetic retinopathy; NPDR, nonproliferative diabetic retinopathy; PDR, proliferative diabetic retinopathy.

The central 1 mm zone exhibited between‐group differences. The 3–6 mm region displayed selective involvement, with nasal quadrant density in patients with DM being significantly lower than that in controls but higher than that in patients with PDR.

### 3.3. Macular GCC Thickness of the Four Groups

GCC measurements revealed nonlinear neurodegenerative patterns (Table 3). Global thinning was more severe in patients with PDR than in both controls and patients with NPDR. Sectoral analysis identified the superonasal region as the most vulnerable, with PDR values 7.8% lower than those of NPDR. The minimum GCC thickness showed a dramatic reduction in the PDR compared with all other groups.

**Table 3:** Macular GCC thickness measurements across study groups.

**Parameter**	**Healthy controls (** **n** = 49**)**	**DM without DR (** **n** = 53**)**	**NPDR (** **n** = 55**)**	**PDR (** **n** = 55**)**	**Statistics**	**p** **value**	**Post hoc comparisons**
Average GCC thickness (*μ*m)	79.51 ± 6.56	76.41 ± 7.39	80.62 ± 8.34	72.73 ± 13.89	*F* = 6.522	< 0.0001	c,f
Superotemporal GCC thickness (*μ*m)	78.63 ± 6.62	76.97 ± 10.15	80.37 ± 10.96	72.94 ± 19.49	*F* = 2.975	0.033	f
Superior GCC thickness (*μ*m)	80.35 ± 6.77	76.53 ± 8.97	82.66 ± 10.25	72.92 ± 19.70	*F* = 5.534	0.001	c,f
Superonasal GCC thickness (*μ*m)	80.70 ± 7.09	75.93 ± 8.40	85.36 ± 11.53	74.41 ± 14.62	*F* = 9.499	< 0.0001	c,d,f
Inferonasal GCC thickness (*μ*m)	78.88 ± 7.47	75.58 ± 8.38	79.63 ± 9.08	73.25 ± 15.37	*F* = 3.664	0.014	f
Inferior GCC thickness (*μ*m)	78.19 ± 7.07	74.73 ± 7.97	77.24 ± 9.54	70.72 ± 16.06	*F* = 4.439	0.005	c,f
Inferotemporal GCC thickness (*μ*m)	80.25 ± 6.87	78.39 ± 8.96	78.14 ± 13.12	70.60 ± 17.85	*F* = 5.223	0.002	c,f
Minimum GCC thickness (*μ*m)	39.70 ± 11.24	35.19 ± 10.59	31.42 ± 16.44	15.62 ± 15.84	*F* = 24.546	< 0.0001	b,c,e,f

*Note:* Post hoc comparisons: a = healthy vs. DM, b = healthy vs. NPDR, c = healthy vs. PDR, d = DM vs. NPDR, e = DM vs. PDR, f = NPDR vs. PDR (all *p* < 0.05, Bonferroni′s correction).

Abbreviations: DM, diabetes mellitus; DR, diabetic retinopathy; GCC, ganglion cell complex; NPDR, nonproliferative diabetic retinopathy; PDR, proliferative diabetic retinopathy.

### 3.4. Conjunctival VD of the Four Groups

Conjunctival VD paralleled the retinal changes (Table 4). Temporal conjunctival VD decreased stepwise from control to PDR. Nasal conjunctival VD showed similar trends but with smaller effect sizes. No significant difference was observed between the controls and patients with DM without DR in either region.

**Table 4:** Conjunctival VD across study groups.

**Parameter**	**Healthy controls (** **n** = 49**)**	**DM without DR (** **n** = 53**)**	**NPDR (** **n** = 55**)**	**PDR (** **n** = 55**)**	**Statistics**	**p** **value**	**Post hoc comparisons**
Temporal conjunctival VD (%)	70.67 ± 6.57	68.18 ± 9.29	58.93 ± 10.02	55.61 ± 9.29	*F* = 29.802	< 0.0001	b,c,d,e
Nasal conjunctival VD (%)	67.18 ± 6.39	67.04 ± 9.39	58.45 ± 9.75	57.89 ± 10.09	*F* = 14.170	< 0.0001	b,c,d,e

*Note:* Post hoc comparisons: a = healthy vs. DM, b = healthy vs. NPDR, c = healthy vs. PDR, d = DM vs. NPDR, e = DM vs. PDR, f = NPDR vs. PDR (all *p* < 0.05, Bonferroni′s correction).

Abbreviations: DM, diabetes mellitus; DR, diabetic retinopathy; NPDR, nonproliferative diabetic retinopathy; PDR, proliferative diabetic retinopathy; VD, vessel density.

### 3.5. Interparameter Correlation Analysis

**Figure 2:**
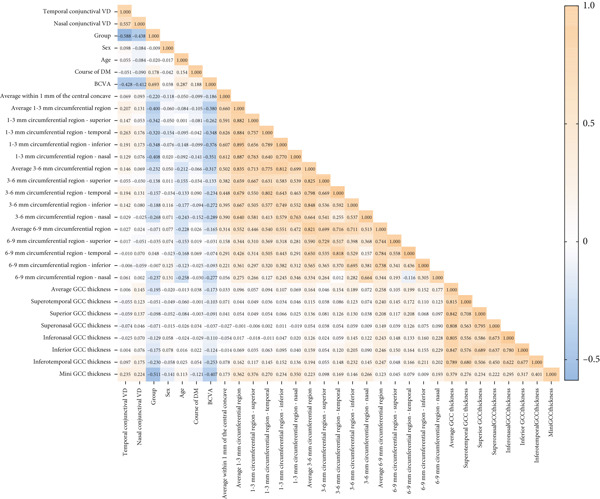
Correlation heatmap of ocular parameters in diabetic retinopathy progression. Heatmap visualization of Spearman′s correlation coefficients among conjunctival, retinal, and neuroretinal parameters in diabetic retinopathy (DR). Color gradient represents correlation strength (red: positive; blue: negative), with asterisks (∗) indicating statistically significant correlations ( ^∗^ *p* < 0.05 and  ^∗∗^ *p* < 0.01). Parameters are grouped by anatomical region (conjunctival, retinal vascular, and GCC). The clustering pattern reveals three distinct correlation modules: (1) conjunctival microvasculature (temporal/nasal VD), (2) retinal vascular density (1–3/3–6 mm zones), and (3) neuroretinal structural parameters (GCC thickness). BCVA, best corrected visual acuity; GCC, ganglion cell complex; VD, vascular density.

The comprehensive correlation matrix (Figure 2) revealed significant interrelationships among the conjunctival, retinal, and neural parameters across the DR stages. Temporal and nasal conjunctival VD showed a strong positive correlation (*r* = 0.557, *p* < 0.01). Both conjunctival measures negatively correlated with DR severity (group: *r* = −0.588 temporal, *r* = −0.438 nasal, *p* < 0.01). Temporal conjunctival VD demonstrated particularly strong inverse correlations with clinical DR stage (*r* = −0.59, *p* < 0.01), BCVA (*r* = −0.43, *p* < 0.01), and retinal vascular density in the 1–3 mm zone (*r* = 0.21–0.26, *p* < 0.01). The nasal conjunctival measurements showed generally weaker (but still significant) correlations with these same parameters (group: *r* = −0.44; BCVA: *r* = −0.41). This temporal–nasal difference was consistent across nearly all analyses. Both conjunctival measures correlated with minimum GCC thickness, though the temporal VD again showed stronger associations (*r* = 0.22 vs. 0.17 for nasal).

## 4. Discussion

Our study revealed a progressive decline in conjunctival VD with advancing DR, with the temporal conjunctival region showing the most significant changes. Conjunctival VD correlates with both retinal vascular parameters and structural alterations in the neural retina. These findings suggest that SS‐OCTA assessment of conjunctival VD, particularly in the temporal quadrant, could serve as a valuable alternative for evaluating the retinal microvascular status in DR patients with poor fundus visibility.

Owing to its superficial location, excellent optical accessibility, and high‐resolution imaging potential, the conjunctival microcirculation provides an ideal window for assessing systemic microvascular pathology. Unlike fundus examination, conjunctival vessel imaging does not require pupil dilation and is unaffected by refractive media opacities. Previous studies utilizing imaging techniques, including AS cameras, computer‐assisted in vivo microscopy, and functional slit‐lamp biomicroscopy, have demonstrated that bulbar conjunctival vascular width and tortuosity increase with DR severity, whereas overall vascular density decreases in patients with diabetes [4, 14, 15]. SS‐OCTA provides higher vascular density quantification than slit‐lamp examination across all quadrants, with its depth‐resolved imaging capability, allowing targeted vascular assessment of the conjunctiva [4, 5, 16].

Our findings align with those reported by Schuerch et al. [12], although the absolute values of conjunctival VD differed between the studies. This discrepancy may be attributed to the differences in imaging systems and analytical protocols. Specifically, we defined the conjunctival vascular measurement boundary as extending from the superficial conjunctival layer to the anterior border of the scleral stroma, with the final measurements representing the average values across this region. In contrast, Schuerch et al. utilized Heidelberg Spectralis spectral‐domain OCT in the OCTA mode, adapted for AS imaging by coupling a 25D lens with a standard 30° fundus lens. Their acquisition protocol involved 10^°^ × 5^°^ cube scans centered at the limbus in both the temporal and nasal conjunctival quadrants, with subsequent binarization and analysis using ImageJ (Fiji Version 2.0) to determine the average relative conjunctival VD [12]. However, they did not perform subgroup analyses stratified by DR. To investigate disease stage dependency, we systematically grouped participants according to clinically validated DR severity scales, which revealed progressive microvascular changes with advancing retinopathy.

Temporal conjunctival VD showed a progressive reduction from the control (70.67%) to PDR (55.61%). No significant conjunctival VD differences existed between controls and patients with diabetes without DR, suggesting a specificity for retinopathic changes rather than systemic diabetes. Temporal conjunctival VD demonstrated stronger correlations with DR severity (*r* = −0.588) than did nasal assessments (*r* = −0.438), indicating regional diagnostic utility. Comprehensive correlation analysis revealed significant positive associations between temporal conjunctival VD and both retinal VD (*r* = 0.21–0.26) and GCC thickness (*r* = 0.22) and inverse correlations with clinical DR stage (*r* = −0.59) and BCVA (*r* = −0.43). Compared to the nasal conjunctival VD, the temporal conjunctival VD showed the most robust correlations with DR severity, retinal VD, and mini‐GCC thickness, suggesting that this region may be particularly vulnerable to diabetic microangiopathy. A previous OCTA study demonstrated good repeatability and interobserver agreement in assessing limbal vascularization among healthy individuals. While significant differences in vascular density between the temporal and nasal quadrants were reported in that study [17], our findings did not replicate this spatial variation, which is consistent with the findings of Schuerch et al. [12]. This discrepancy may be attributed to methodological differences, particularly in the selection of the region of interest and the anatomical site of measurement (limbus vs. conjunctiva). Nonetheless, our analysis maintained a quadrant‐specific approach to evaluate the nasal and temporal regions independently to ensure a comprehensive assessment.

Diabetes progression induces vasoconstriction in the bulbar conjunctival microvasculature, resulting in decreased total vascular area relative to that in nondiabetic individuals [6, 18]. However, our data do not corroborate this observation. Notably, no significant differences in conjunctival VD were found between healthy controls and diabetic patients without DR, implying that these conjunctival microvascular changes are specific to retinopathic progression rather than to systemic diabetes alone. This specificity enhances the clinical utility of conjunctival VD as a potential biomarker of DR.

Retinal OCTA revealed distinct spatial patterns of vascular compromise. The 1–3 mm parafoveal region exhibited the most marked vascular attenuation, particularly in the nasal quadrant (63.79% in controls vs. 42.07% in patients with PDR). Early temporal quadrant involvement was observed in patients with NPDR (46.91% vs. 57.99% in controls), suggesting that this region may serve as a sentinel marker of initial microvascular damage. The central 1 mm zone showed significant VD reduction across all diabetic groups, indicating early foveal microcirculation compromise, regardless of retinopathy status.

GCC thickness measurements demonstrated nonlinear neurodegenerative changes. Average GCC thickness was most pronounced in PDR (72.73 vs. 79.51 *μ*m in controls). Sectoral analysis identified the superonasal GCC as particularly vulnerable (85.36 *μ*m in NPDR vs. 74.41 *μ*m in PDR), with 12.8% relative thinning. The minimum GCC thickness showed a dramatic reduction in PDR (15.62 vs. 39.70 *μ*m in controls), highlighting focal neurodegeneration in advanced disease.

Our study demonstrated that microvascular impairment in diabetes is a systemic process that affects multiple vascular beds. Conjunctival microvascular assessments provide a unique window for monitoring diabetic microvascular health. Importantly, our data revealed a progressive reduction in conjunctival VD, which significantly correlated with decreased retinal vascular density and thinning of the GCC. These findings provide compelling evidence for the pathophysiological continuity between the anterior and posterior ocular segment microvascular alterations in DR.

The application of SS‐OCTA to quantify bulbar conjunctival vascular density offers an objective, efficient, and safe approach for evaluating microvascular changes in patients with diabetes. This innovative methodology may establish a more accessible and safer monitoring strategy for patients with DR who cannot undergo conventional fundus examination. Compared to conventional OCTA and fundus examinations, SS‐OCTA‐based conjunctival microvascular density assessment provides several key clinical benefits for diabetic patients. The nonmydriatic and rapid imaging features enhance patient compliance and broaden applicability, particularly in individuals with cataracts or poor fixation. The conjunctival microvascular alterations may precede detectable retinal changes, offering a potential window for earlier intervention in diabetic microvascular complications. Additionally, the high‐resolution quantitative nature of this technology enables precise dynamic monitoring of therapeutic responses while potentially serving as a holistic biomarker for systemic microvascular status. These combined advantages optimize diagnostic workflows and patient management, with the potential to reduce long‐term visual disability and associated healthcare costs through timely intervention.

However, our study had certain limitations. While our sample size was sufficient for initial observations, the cross‐sectional design prevented us from determining causality or predicting progression, and future multicenter longitudinal studies with larger sample sizes are needed. Investigating whether conjunctival changes precede clinically detectable retinopathy would greatly increase the value of screening for conjunctival VD changes.

## 5. Conclusions

In conclusion, our study provides compelling evidence that SS‐OCTA assessment of conjunctival VD, especially in the temporal region, is a promising surrogate marker for retinal pathology in patients with DR and media opacities. Although this approach cannot replace traditional retinal imaging when feasible, it offers a practical alternative for patients with compromised fundus visualization.

## Conflicts of Interest

The authors declare no conflicts of interest.

## Funding

This study was funded by the Suzhou Science and Technology Development Plan Project (SKY2023088), the Key Laboratory Open Topic Funding Project in Jiangsu Universities (XZSYSKF2023020), the Nantong University Clinical Medicine Special Research Fund Project (2024JY029), and the Changshu Science and Technology Program Project (CS202455).

## Data Availability

The raw data supporting the conclusions of this article will be made available by the authors, without undue reservation.
